# Occupational tobacco exposure and multisystem morbidity among beedi rollers: biomarker-validated evidence from a multi-state study in India

**DOI:** 10.3389/fpubh.2026.1799694

**Published:** 2026-04-29

**Authors:** Rakesh Kakkar, Om Prakash Bera, Joanna E. Cohen, Vandana Shah, Monica Kakkar, Sejal Saraf, Surekha Kishore, Sonu Hangma Subba, Chetna Maliye, Darivemula Surendra Babu, Kusum Gaur, Soumya Swaroop Sahoo, Madhur Verma, U. Venkatesh, Binod Kumar Behera, Manish Taywade, Amey Dhatrak, Badal Chandrakant Bhandarkar, Mohnish Yuvrajgir Giri, Varkey Nadakkavukaran Santhosh, Jaspreet Kaur Pal, Radhika Khajuria, Ranjit Singh, Krupal Joshi

**Affiliations:** 1 Department of Community and Family Medicine, All India Institute of Medical Sciences, Bathinda, Punjab, India; 2 Global Health Advocacy Incubator, Washington, DC, United States; 3 Johns Hopkins Bloomberg School of Public Health, Baltimore, MD, United States; 4 Department of Biochemistry, All India Institute of Medical Sciences, Bathinda, Punjab, India; 5 Department of Community and Family Medicine, All India Institute of Medical Sciences, Rishikesh, Uttarakhand, India; 6 Department of Community Medicine and Family Medicine, All India Institute of Medical Sciences, Bhubaneswar, Odisha, India; 7 Department of Community Medicine, Mahatma Gandhi Institute of Medical Sciences, Sewagram, Maharashtra, India; 8 Department of Community Medicine, Employees' State Insurance Corporation Medical College, Sanathnagar, Hyderabad, Telangana, India; 9 Department of Community Medicine, SMS Medical College, Jaipur, India; 10 Department of Community and Family Medicine, All India Institute of Medical Sciences, Gorakhpur, Uttar Pradesh, India; 11 Department of Community Medicine, Government Medical College, Nagpur, Maharashtra, India; 12 Taluka Health Office, Chimur, Maharashtra, India; 13 Campaign for Tobacco-Free Kids, Washington, DC, United States; 14 Supreme Court of India, New Delhi, India; 15 Department of Community and Family Medicine, All India Institute of Medical Sciences, Rajkot, Gujarat, India

**Keywords:** beedi rolling, informal sector, musculoskeletal disorders, occupational health, tobacco exposure, urinary cotinine

## Abstract

**Purpose:**

Beedi rolling, employing nearly five million predominantly female home-based workers across India, remains one of the less formally organized occupational sectors. This study aimed to evaluate the working conditions and health hazards of beedi rollers across five Indian states.

**Methods:**

A multi-centric cross-sectional study conducted in five states enrolled 1,800 adults; 900 beedi rollers and 900 controls. Data were obtained through structured interviews, clinical examinations, spirometry, and biochemical analyses of blood and urine samples. A multivariable binary logistic regression model was used to quantify the adjusted associations between occupational status (beedi rollers vs. non-beedi workers) and various morbidities, accounting for sociodemographic confounders.

**Results:**

Beedi rollers had significantly higher odds of musculoskeletal and exposure-related conditions. Adjusted odds ratios for neck pain, back and leg pain, and lower back pain were 5.77, 3.14, and 1.71, respectively. Respiratory and ocular symptoms were more prevalent, with sneezing (AOR 3.13) and ocular redness (AOR 2.19) markedly elevated. Mean urinary cotinine levels were substantially higher among exposed workers (76.97 ng/ml) compared to controls (15.81 ng/ml; *p* < 0.001), corresponding to an AOR of 4.61 for elevated cotinine. Beedi rollers also exhibited higher diastolic blood pressure and HbA1c, alongside reduced Serum Glutamic-Oxaloacetic Transaminase (SGOT) levels.

**Conclusion:**

This multi-state analysis underscores the profound occupational health burden among beedi rollers, driven by ergonomic strain and chronic tobacco exposure. Despite being a large informal workforce, these workers have yet to be fully integrated into occupational health services, highlighting opportunities to strengthen India's progress toward Universal Health Coverage. Addressing these gaps calls for strengthening workplace design, reinforcing occupational safety standards, and implementing targeted policy interventions.

## 1 Introduction

Tobacco use is one of the leading preventable causes of disease and premature death globally, accounting for over 8 million deaths annually and 229.77 million disability-adjusted life years (DALYs) (1, 2). In India, tobacco use contributes to an estimated 1.35 million deaths each year, with a significant share attributed to beedi smoking (3). Beedis small, indigenous smoking sticks composed of 0.2–0.3 g of sun-dried tobacco flake wrapped in a *Diospyros melanoxylon* (tendu) leaf and tied with colored thread, comprise over 50% of the tobacco market in India (4). A traditional form of hand-rolled tobacco cigarette commonly used in South Asia. Compared to cigarettes, beedis deliver significantly higher levels of nicotine, tar, and carbon monoxide per unit, and tar, compounding health risks for low-income users (5).

The beedi sector is deeply embedded in India's tobacco economy and simultaneously serves as a critical site of informal, home-based employment. Approximately 4.9 million individuals are engaged in beedi rolling, over 90% of whom are women and children (6–8). This occupation is predominantly manual, typically performed at home without mechanization or personal protective equipment, and often under conditions with limited ventilation. Workers often roll 500 to 1,000 beedis per day, handling 225–450 grams of raw tobacco flake (9, 10). This exposure takes place in home settings and communal workplaces where bidi rolling is commonly carried out without protective equipment, often with limited ventilation, thereby increasing the risk of chronic toxicant absorption. Biomonitoring studies show elevated urinary cotinine levels among beedi workers have been consistently observed, highlighting systemic nicotine absorption (11).

The occupational health environment of beedi rolling is characterized by prolonged static postures, repetitive movements, and poor ergonomics (8). Musculoskeletal morbidity, especially shoulder, back, and neck pain, is widely reported (12, 13). Respiratory symptoms, dermatologic irritation, and eye strain are also frequently reported (14). Beedi workers are exposed to nicotine through multiple pathways, including dermal absorption, inhalation of tobacco dust, and hand-to-mouth transfer during handling. A Cross-sectional study by Biswas et al. (15) in West Bengal found significantly lower pulmonary function parameters, measured by PEFR, FEV1, and FVC, among beedi workers compared to controls, with evidence of both obstructive and restrictive lung impairment. This suggests early onset of obstructive and restrictive lung pathologies even among younger cohorts and short-duration workers.

Under India's Occupational Safety, Health and Working Conditions (OSH) Code, 2020, beedi rolling has not yet been classified as a hazardous occupation, presenting an opportunity for evidence-based policy revision (16). Further, changes in policy frameworks, including transitions in the Beedi Workers Welfare Fund Act (1976), have created gaps in access to statutory entitlements and occupational health services, highlighting the need for updated protective mechanisms (17). This situation reflects broader challenges within India's informal economy, which encompasses over 74% of the workforce and over 80% of working women (18). The beedi industry exemplifies these vulnerabilities. It operates in the gray zone between the organized and unorganized sectors, with production decentralized across subcontractor chains and lacking formal employer-employee contracts (19). Workers in this sector frequently face challenges including irregular income, quality disputes over finished products, limited job security, and gaps in access to welfare programs (8, 20).

From a socioeconomic perspective, beedi workers earn approximately ₹,000–3,000 per month, with income subject to variability due to irregular work and quality-based assessments, placing many households in economically vulnerable circumstances (21). Women workers in particular face higher levels of anemia, gynecological morbidity, and report adverse maternal-child health outcomes, including stunting and low birth weight (22, 23). Compounding these material hardships is a general lack of awareness among workers regarding the long-term health impacts of beedi rolling, particularly respiratory impairment and toxic exposure (9).

Although prior studies have highlighted various health risks associated with beedi rolling, much of the existing literature remains fragmented, often geographically limited, based on small sample sizes, or lacking integration of clinical, occupational, and socioeconomic dimensions (8, 15). There remains a critical gap in nationally relevant, cross-sectional evidence that draws on diverse regional data, incorporates biomarker and respiratory assessments, and systematically evaluates working conditions and health outcomes in this vulnerable workforce. Given the high levels of occupational exposure, socioeconomic vulnerability, and limited representation of beedi workers in existing research and policy discourse, there is a pressing public health need to generate comprehensive and generalizable evidence to inform targeted interventions and policy action.

Furthermore, few studies contextualize the health risks within the broader structural and policy landscape, including the evolving labor welfare legislative framework and the current occupational classification status of beedi rolling. Addressing this evidence gap is essential for supporting occupational classification decisions, intervention design, and alignment with international commitments under the WHO Framework Convention on Tobacco Control (WHO-FCTC) (24).

The present study explores the health hazards and livelihood-related problems among beedi rollers across five geographically diverse states in India. This study is among the few multi-state investigations in India to comprehensively integrate objective biomarkers, spirometry, and socioeconomic assessments to evaluate the multidimensional health burden among beedi workers. The study aims to assess the health hazards and working conditions of beedi rollers, and to compare their morbidity profile and associated factors with those of non-beedi rollers. This large-scale, multi-regional study simultaneously captures the clinical, occupational, and socioeconomic vulnerabilities of beedi workers across India's heterogeneous labor landscape. The findings have the potential to directly inform the evidence-based recognition of beedi rolling as a hazardous occupation, the design of targeted occupational health interventions, and the reinstatement or reform of social protection mechanisms for this marginalized workforce.

## 2 Methods

### 2.1 Study design

**Figure 1:**
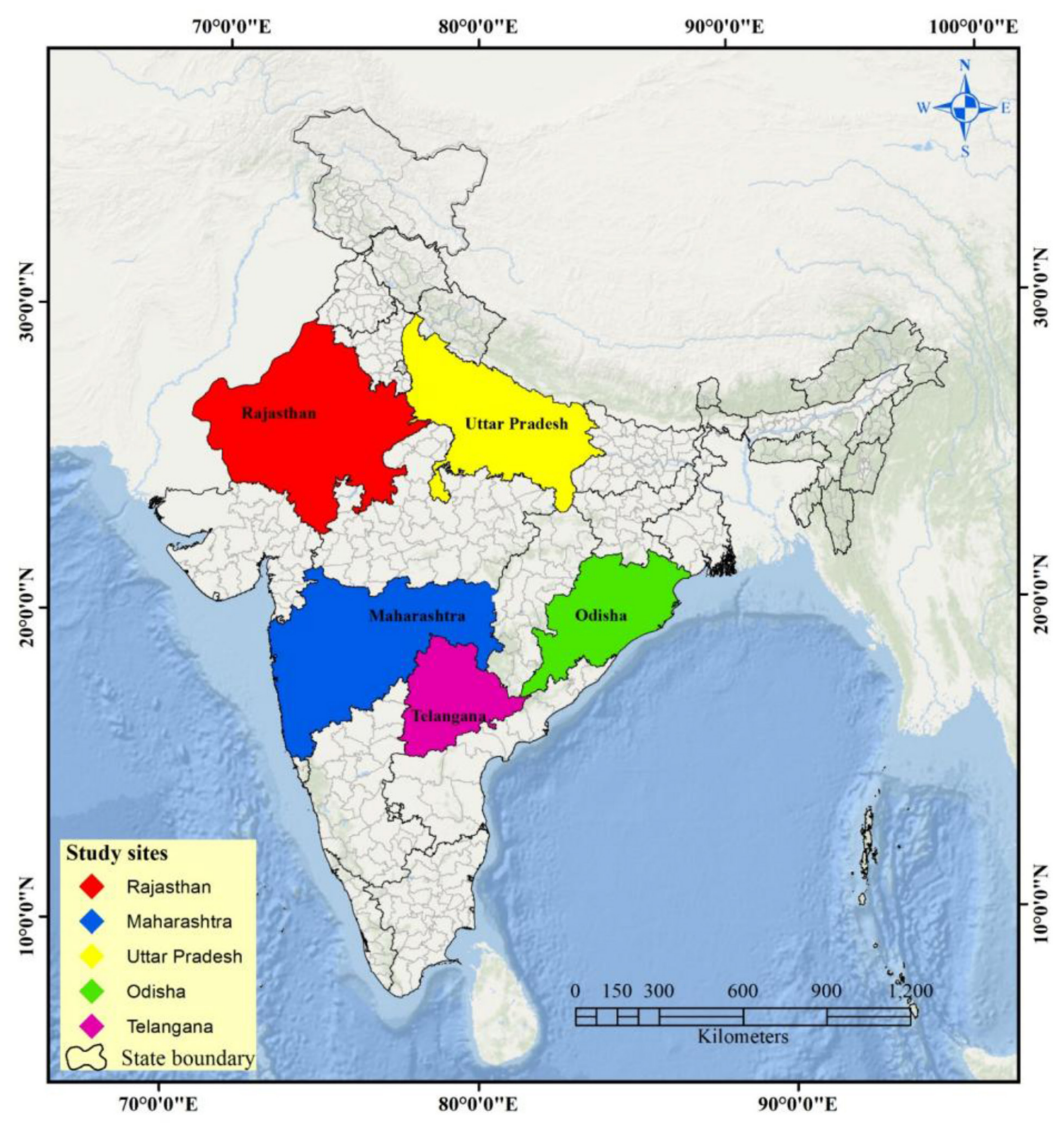
Geographical distribution of study sites across five Indian states involved in the beedi rollers study.

We employed a cross-sectional study design with retrospective assessment of self-reported occupational exposures and associated health outcomes among beedi rollers and a control group of non-beedi rollers. The study adhered to the Strengthening the Reporting of Observational Studies in Epidemiology (STROBE) guidelines. The study was conducted across five Indian states: Uttar Pradesh (North), Maharashtra and Rajasthan (West), Telangana (South), and (East), selected based on high concentration of registered beedi workers, as identified through International Labor Organization (ILO) data (25). Figure 1 shows the geographical distribution of the study sites. The study design was reviewed and approved by the Institutional Ethics Committee at the All-India Institute of Medical Sciences (AIIMS), Bhatinda. The protocol was also approved by the Johns Hopkins Bloomberg School of Public Health Institutional Review Board. Written informed consent was obtained from all participants before data collection, in accordance with the approved institutional ethics protocol.

### 2.2 Study setting

Data collection was conducted between August 2023 to October 2024, in purposively selected districts within each of the five states. One district with a high density of registered beedi workers was selected in each state. From each district, two villages were identified, one predominantly engaged in beedi rolling (exposed group) and one with no known beedi-rolling activity (unexposed group). Structured interviews and clinical assessments were conducted in household settings, in participant preferred languages: Hindi, English, or Telugu.

Participants were selected using a multi-stage stratified sampling strategy. In each state, the five districts with the highest number of registered beedi-roller (per ILO data) were identified (25). One district per state was chosen in consultation with local experts. From each district, one beedi-rolling and one non-beedi-rolling village were selected. Recruitment involved door-to-door household visits, with one eligible adult selected per household using a random-start method based on the next birthday of an adult household member.

### 2.3 Study participants

We enrolled adults aged ≥18 years residing in selected villages across the five states. Participants were categorized into two groups based on occupational exposure. The exposed group (beedi rollers) comprised participants continuously engaged in beedi rolling for ≥12 months prior to enrolment, while the unexposed group (non-beedi rollers) included participants never engaged in beedi rolling and were employed in alternative occupations for ≥12 months. Eligibility required continuous residence in the village for ≥1 year, ability to communicate in Hindi, English, or Telugu, and availability during data collection. Exclusion criteria included: age < 18 years, language/scheduling constraints, or cognitive limitations. Vulnerable populations, including children and cognitively impaired individuals, were not included.

### 2.4 Study variables

The primary outcomes included the prevalence of self-reported and clinically assessed respiratory symptoms, musculoskeletal disorders, and other morbidity indicators associated with beedi rolling. Exposure was defined as occupational involvement in beedi rolling over the past 12 months. Clinical biomarkers and relevant covariates were analyzed as binary variables based on standard thresholds. Variable definitions, coding schemes, and measurement details are provided in Table 1 to ensure consistency across all study sites.

**Table 1:** Variables used in the study: definitions, categories, and measurement methods.

Variable	Type	Category/cut-off	Measurement method
Dependent variable			
15.6-7.4,-14499ptBeedi rollers vs. Non-beedi rollers	Dichotomous	Yes = 1, No = 2	Self-reported occupational exposure in past 12 months
Independent variables			
Clinical/Biochemical Indicators			
Cotinine (nicotine exposure)	Dichotomous	Elevated (≥ 44.5 ng/mL) = 1, Normal = 0	Urine sample (10 mL midstream); lab-tested
Creatinine (renal function)	Dichotomous	Abnormal [> 1.35 mg/dL (men) or > 1.04 mg/dL (women)] = 1, Normal = 0	Blood test; lab-analyzed
SGOT (liver enzyme)	Dichotomous	Abnormal [> 48 U/L (men) or > 43 U/L (women)] = 1, Normal = 0	Blood test; lab-analyzed
GGT	Dichotomous	Abnormal (> 40 U/L) = 1, Normal = 0	Blood test; lab-analyzed
Total protein	Dichotomous	Low (< 6.0 g/dL) = 1, Normal = 0	Blood test; lab-analyzed
HbA1c (glycemic control)	Dichotomous	Poor control (≥ 6.5%) = 1, Normal = 0	Blood test; lab-analyzed
Hemoglobin (anemia)	Dichotomous	Low (< 13.0 g/dL in men or < 12.0 g/dL in women) = 1, Normal = 0	Blood test; lab-analyzed
Waist-to-hip ratio	Dichotomous	High Risk (> 0.90 for men; > 0.85 for women) = 1, Normal = 0	Measured using tape; calculated post-measurement
15.6-7.4,-14499ptPulmonary Function Test (PFT)	Dichotomous	Abnormal = 1 (FEV1/FVC < 0.70 per GOLD criteria), Normal = 0	Spirometry: FEV1, FVC, FEV1/FVC, PEFR
Musculoskeletal problems			
15.6-7.4,-14499pt(neck, elbow, back, knee, leg, spinal combination)	Dichotomous	Yes = 1, No = 2	Self-reported
Respiratory problems			
15.6-7.4,-1499pt (cough, sneezing)	Dichotomous	Yes = 1, No = 2	Self-reported
Ocular problems			
15.6-7.4,-1499pt (decreased vision, watering, redness)	Dichotomous	Yes = 1, No = 2	Self-reported
Symptoms			
15.6-7.4,-1499pt(weakness, breathlessness, palpitations)	Dichotomous	Yes = 1, No = 2	Self-reported
Dermatological problems			
(hand pigmentation, itching)	Dichotomous	Yes = 1, No = 2	Self-reported
Chronic disease	Dichotomous	Yes = 1, No = 2	Self-reported

PFT, Pulmonary Function Test; FEV1, Forced Expiratory Volume in 1 second; FVC, Forced Vital Capacity; PEFR, Peak Expiratory Flow Rate.

### 2.5 Data sources and measurement

Data were collected using structured, pretested interviewer-administered questionnaires, followed by clinical examinations. The questionnaire covered sociodemographic characteristics, occupational history, lifestyle factors, housing and sanitation, and self-reported morbidity. Clinical assessments included respiratory and musculoskeletal health evaluations, conducted under uniform protocols to reduce inter-observer variability. Spirometry assessments were conducted following standard quality control guidelines, with repeated trials performed to ensure accuracy and reliability of measurements.

Biochemical data were derived from blood and urine samples collected during clinical assessment. Variables such as cotinine, creatinine, serum glutamate oxaloacetate transaminase (SGOT), gamma-glutamyl transferase (GGT), total protein, hemoglobin A1c (HbA1c), hemoglobin, and waist-to-hip ratio were assessed using standardized laboratory methods and categorized according to clinical thresholds (see Table 1).

All field personnel were trained using the Johns Hopkins Bloomberg School of Public Health Human Subjects Research Ethics Field Training Guide. The comparability of data collection methods was ensured by standardizing procedures across all states and continuous monitoring by state-specific research associates.

### 2.6 Sample technique and bias control

To reduce selection bias, we employed a multistage stratified sampling design, wherein districts were selected based on high beedi worker density, followed by the selection of one exposed and one unexposed village within each district, and random household selection. Information bias was mitigated through interviewer training, use of validated survey instruments, and standardized clinical protocols. Age and gender matching between groups minimized confounding. Additionally, clinical assessments were conducted alongside participant responses, providing an objective measure to complement self-reported information.

### 2.7 Study size

Sample size was calculated using the equation:


$$\begin{array}{l} n=\frac{4_{pq}}{l^{2}} \end{array}$$

Where *p* = 27% (prevalence of respiratory symptoms among beedi rollers) (26), q = 100 – *p* = 73%, and l = 5% (absolute error). Accounting for a 10% non-response rate, we required a minimum of 346 participants per state, resulting in a total requirement of 1,730 participants across five states. To ensure adequate representation and account for any unforeseen exclusions, a total of 1,800 adults were ultimately enrolled: 900 beedi rollers and 900 matched non-beedi workers, maintaining a 1:1 ratio between exposed and unexposed groups.

### 2.8 Statistical methods

Descriptive statistics were used to summarize demographic, occupational, and health-related variables. Quantitative variables were analyzed as either continuous (e.g., hours worked per day) or categorical (e.g., symptoms presence/absence). For analysis, continuous variables such as cotinine, creatinine, SGOT, GGT, total protein, HbA1c, hemoglobin, waist-to-hip ratio, and pulmonary function test were categorized using clinically relevant thresholds. Chi-square (χ^2^) tests compared categorical variables between groups, while the Mann-Whitney U test was used for continuous variables not normally distributed. A significance level of *p* ≤ 0.05 was considered for all statistical tests.

Binary Logistic regression model (BLRM) was employed to examine the association between occupational status (beedi rollers vs. non-beedi rollers) and various health conditions. Occupational status was modeled as the primary exposure variable to estimate its independent effect on multiple health outcomes, which were treated as dependent variables in separate regression models, adjusted for age, gender, education, and occupation. BLRM estimates the probability of an event occurring (e.g., presence of a health problem) based on multiple predictor variables, which may be categorical or continuous. In a BLRM, the model's structure is represented by equation:


$$\begin{array}{l} log\;\left(\frac{pi}{1-pi}\right)=\beta_{0}+\beta_{1}x_{1},i+\ldots +\beta_{n}x_{n},i \end{array}$$

where *p*_*i*_is the probability that the *i*^*th*^ individual is a beedi rollers (coded as 1), *x* represents the independent variables (potential health-related factors), and β denotes the estimated coefficient.

A forward stepwise selection method was used to build the most parsimonious model, where variables were added one at a time based on their statistical significance (typically at *p* < 0.05 or 0.10) and contribution to model performance, assessed using the Akaike Information Criterion (AIC) and likelihood ratio tests. This process continued until no further statistically significant variables improved the model. All the regression models were adjusted for age, gender, education, and occupation. Although a forward stepwise approach was used for model selection, its inherent limitations, including potential overfitting and instability of variable selection, are acknowledged.

Model fit was evaluated using the Hosmer-Lemeshow goodness-of-fit test (27), −2 Log Likelihood (28), Cox and Snell R^2^ (29), and Nagelkerke R^2^ statistics (28). Odds Ratios (ORs), Adjusted Odds Ratios (AORs) with 95% Confidence Intervals (CIs), and associated *p*-values were calculated to interpret the direction and strength of associations. Variables with *p* < 0.05 were considered statistically significant.

## 3 Results

### 3.1 Demographic characteristics of study participants

Table 2 presents the demographic distribution of beedi rollers and non-beedi rollers. A significantly higher proportion of females were involved in beedi rolling (95.4%). The majority of beedi rollers belonged to the older adult age group (48.1%), whereas non-beedi workers were more evenly distributed across age categories. Educational attainment was generally lower among beedi rollers, with 28.8% having no formal schooling compared to 32.7% among non-beedi rollers; however, the proportion with graduate-level education was substantially lower among beedi rollers (3.2%) than their counterparts (12.0%). Non-beedi rollers were distributed across diverse occupations including agriculture (19.9%), daily wage labor (34.9%), and government jobs (6.9%).

**Table 2:** Comparison of demographic characteristics of the study participants.

Demographic variables	Beedi rollers *n* = 900 (%)	Non-beedi rollers *n* = 900 (%)
Gender	
Male	41 (4.6%)	60 (6.7%)
Female	859 (95.4%)	840 (93.3%)
Age categories	
Young adults	137 (15.2%)	179 (19.9%)
Middle aged adults	330 (36.7%)	359 (39.9%)
Old aged adults	433 (48.1%)	362 (40.2%)
Education	
No formal schooling	259 (28.8%)	294 (32.7%)
Primary school	345 (38.3%)	266 (29.6%)
Secondary school	210 (23.3%)	197 (21.9%)
Undergraduate	57 (6.3%)	35 (3.9%)
Graduate	29 (3.2%)	108 (12.0%)
Occupation	
Government employee	0 (0%)	62 (6.9%)
Agriculture	0 (0%)	179 (19.9%)
Daily wage laborer	0 (0%)	314 (34.9%)
Business owner	0 (0%)	63 (7.0%)
Beedi rolling	900 (100%)	0 (0%)
Homemaker	0 (0%)	265 (29.4%)
Unemployed	0 (0%)	3 (0.3%)
Retired	0 (0%)	14 (1.6%)
Marital status	
Never married	42 (4.7%)	41 (4.6%)
Married	725 (80.6%)	741 (82.3%)
Separated	4 (0.4%)	7 (0.8%)
Divorced	3 (0.3%)	3 (0.3%)
Widow/Widower	126 (14.0%)	108 (12.0%)
Total	900 (100%)	900 (100%)

All values are expressed in frequencies with percentage (in parenthesis).

### 3.2 Workplace environmental characteristics among beedi rollers and non-beedi rollers

A significant disparity was observed in workplace environmental conditions between beedi rollers and non-beedi rollers (Table 3). The majority of beedi rollers (94.3%) worked inside their homes, predominantly in living rooms (77.2%), whereas non-beedi rollers primarily worked outside the house (81.6%) or in kitchens (21.7%) when indoors, with these differences being significant (χ^2^ = 1,056.65 and χ^2^ = 1,490.67; *p* < 0.001). Notably, a substantial proportion of beedi rollers (14.0%) reported lack of cross ventilation at their workplace, in contrast to only 0.6% among non-beedi rollers (χ^2^ = 97.96, *p* < 0.001),. Among the 900 beedi rollers, the average number of beedis rolled per day was 654.89 ± 253.17, and the average number of hours spent on beedi rolling per day was 5.74 ± 2.12.

**Table 3:** Distribution between beedi roller status and workplace environment characteristics (*N* = 1,800).

Variable	Beedi rollers (*n* = 900)	Non-beedi rollers (*n* = 900)	χ^2^	*p*-value
Place of work				
Inside the house	849 (94.3%)	165 (18.3%)	1,056.65	< 0.001^*^
Outside the house	51 (5.7%)	734 (81.6%)		
On-site	0 (0.0%)	1 (0.1%)		
Location inside house				
Living room	695 (77.2%)	5 (0.7%)	1,490.67	< 0.001^*^
Kitchen	1 (0.1%)	156 (21.7%)		
Separate room	143 (15.9%)	4 (0.6%)		
Others	38 (4.2%)	0 (0.0%)		
Cross ventilation				
Yes	774 (86.0%)	716 (99.4%)	97.96	< 0.001^*^
No	126 (14.0%)	4 (0.6%)		
Adequate lighting				
Yes	887 (98.6%)	717 (99.6%)	4.32	0.038^*^
No	13 (1.4%)	3 (0.4%)		

All values are expressed in frequencies with percentage (in parenthesis). Statistical test used: Chi-Square test; ^*^P ≤ 0.05 is considered statistically significant.

### 3.3 Comparative analysis of self-reported morbidities in beedi rollers and non-beedi rollers

Table 4 reveals significant health disparities between beedi rollers and non-beedi rollers across multiple health conditions. Beedi rollers report substantially higher prevalence of musculoskeletal problems, particularly back and leg pain (79.4% vs. 50.9%), neck pain (59.3% vs. 23.8%), and low back pain (71.0% vs. 53.0%), all with *p* < 0.001, indicating statistically significant associations with occupational exposure. Ocular problems are also more common among beedi rollers, especially decreased vision (54.7% vs. 42.0%), watering of eyes (27.1% vs. 11.0%), and redness (13.3% vs. 4.1%), each showing strong significance (*p* < 0.001). Respiratory complaints such as difficulty in breathing (13.7% vs. 8.4%) and sneezing (13.8% vs. 4.4%) were also notably higher among beedi rollers (*p* < 0.001). Moreover, dermatological issues like pigmentation, itching, and wounds on hands showed significantly higher rates in the beedi rolling group (*p* < 0.001). Among chronic diseases, hypertension (30.4% vs. 19.3%) was markedly more prevalent in beedi rollers (*p* < 0.001).

**Table 4:** Distribution between beedi roller status and self-reported health problems (*N* = 1,800).

Variable	Beedi rollers (*n* = 900)	Non-beedi rollers (*n* = 900)	χ^2^	*p*-value
Musculoskeletal problems				
*Neck pain*	534 (59.3%)	214 (23.8%)	234.24	< 0.001^*^
*Shoulder joint pain*	345 (38.3%)	245 (27.2%)	25.21	< 0.001^*^
*Elbow joint pain*	169 (18.8%)	161 (17.9%)	0.24	0.626
*Wrist joint pain*	214 (23.8%)	201 (22.3%)	0.53	0.467
*Low Back pain*	639 (71.0%)	477 (53.0%)	61.89	< 0.001^*^
*Knee Joint pain*	523 (58.1%)	461 (51.2%)	8.62	0.003^*^
*Back and leg pain*	715 (79.4%)	458 (50.9%)	161.65	< 0.001^*^
*Spinal pain*	285 (31.7%)	256 (28.5%)	2.18	0.140
*Combination*	236 (26.2%)	191 (21.2%)	6.22	0.013^*^
Respiratory problems				
*Cough with sputum*	20 (2.2%)	48 (5.3%)	11.982	0.001^*^
*Dry cough*	36 (4.0%)	35 (3.9%)	0.015	0.904
*Difficulty breathing*	123 (13.7%)	76 (8.4%)	12.480	< 0.001^*^
*Chest tightness*	71 (7.9%)	70 (7.8%)	0.008	0.930
*Sore throat*	14 (1.6%)	15 (1.7%)	0.035	0.851
*Nasal congestion*	17 (1.9%)	17 (1.9%)	0.000	1.000
*Sneezing*	124 (13.8%)	40 (4.4%)	47.34	< 0.001^*^
Ocular problems				
*Ocular problem*	462 (51.3%)	255 (28.3%)	99.33	< 0.001^*^
*Decreased vision*	492 (54.7%)	378 (42.0%)	28.91	< 0.001^*^
*Watering from the eyes*	244 (27.1%)	99 (11.0%)	75.73	< 0.001^*^
*Redness of eyes*	120 (13.3%)	37 (4.1%)	48.07	< 0.001^*^
Dermatological problems				
Pigmentation of hands	116 (12.9%)	48 (5.3%)	31.02	< 0.001^*^
Itching of hands	78 (8.7%)	23 (2.6%)	31.73	< 0.001^*^
Wounds over hands	31 (3.4%)	8 (0.9%)	13.87	< 0.001^*^
Health symptoms				
*Weakness*	511 (56.8%)	520 (57.8%)	0.18	0.668
*Easy fatigability*	150 (16.7%)	102 (11.3%)	10.63	0.001^*^
*Breathlessness on exertion*	103 (11.4%)	79 (8.8%)	3.521	0.061
Palpitation	47 (5.2%)	12 (1.3%)	21.466	< 0.001^*^
*Giddiness*	113 (12.6%)	91 (10.1%)	2.65	0.104
Chronic disease				
Hypertension	274 (30.4%)	174 (19.3%)	29.72	< 0.001^*^
Diabetes mellitus	61 (6.8%)	53 (5.9%)	0.60	0.439
Ischemic heart disease	7 (0.8%)	3 (0.3%)	1.61	0.205
Bronchial asthma	8 (0.9%)	8 (0.9%)	0.00	1.000
Goiter	3 (0.3%)	2 (0.2%)	0.201	0.654
*Tuberculosis*	5 (0.6%)	4 (0.4%)	0.11	0.738
*Asthma*	23 (2.6%)	23 (2.6%)	0.00	1.000
Cancer	1 (0.1%)	0 (0.0%)	1.001	0.317
Combination	0 (0.0%)	2 (0.2%)	1.978	0.160

All values are expressed in frequencies with percentage (in parenthesis). Statistical test used: Chi-Square test; ^*^ *P* ≤ 0.05 is considered statistically significant.

Table 5 presents a comparative analysis of vital health parameters between beedi rollers and non-beedi rollers, revealing several statistically significant differences. Beedi rollers exhibited significantly lower waist-to-hip ratio (0.89 vs. 0.94, *p* < 0.001). Blood pressure profiles showed higher diastolic values among beedi rollers (*p* < 0.001), suggesting early cardiovascular stress. Cotinine levels, a biomarker of tobacco exposure, were markedly elevated in beedi rollers (76.97 ng/ml vs. 15.81 ng/ml, *p* < 0.001), validating occupational exposure. Biochemical analysis indicated significantly lower serum creatinine (*p* < 0.001), SGOT (*p* < 0.001), and globulin (*p* < 0.001), while albumin, albumin/globulin ratio, and total protein were higher, suggesting altered liver and protein metabolism. Additionally, GGT, HbA1c, and random blood sugar levels were significantly elevated, indicating potential early metabolic disruptions.

**Table 5:** Comparison of metabolic and biochemical health parameters of the study participants.

Health parameters	Beedi rollers (*n* = 900)	Non-beedi rollers (*n* = 900)	Z	*P*-value
Body mass index	23.52 ± 5.45	24.21 ± 6.69	−1.48	0.139
Waist to hip ratio	0.89 ± 0.07	0.94 ± 0.07	−12.65	0.001^*^
Systolic blood pressure	127.10 ± 22.45	125.10 ± 17.30	−0.49	0.625
Diastolic blood pressure	83.16 ± 11.62	81.15 ± 10.06	−3.86	0.001^*^
Hemoglobin	11.89 ± 1.51	11.65 ± 1.56	−3.61	0.001^*^
Lung function (FEV1/FVC ratio)	0.77 ± 0.12	0.80 ± 0.12	−1.77	0.077
Cotinine levels	76.97 ± 90.50	15.81 ± 24.72	−24.49	0.001^*^
Creatinine levels	0.75 ± 0.25	0.85 ± 0.38	−11.12	0.001^*^
Uric acid	4.30 ± 1.20	4.24 ± 1.21	−1.26	0.209
Triglycerides	140.75 ± 91.48	140.85 ± 90.13	−0.178	0.858
Total cholesterol	184.54 ± 39.20	182.54 ± 41.17	−1.13	0.260
LDL	109.16 ± 33.09	106.24 ± 35.87	−1.35	0.176
HDL	47.25 ± 10.21	46.73 ± 8.05	−0.26	0.796
Albumin	4.50 ± 0.33	4.42 ± 0.32	−5.42	0.001^*^
Globulin	3.26 ± 0.50	3.58 ± 0.57	−13.39	0.001^*^
Albumin/globulin ratio	1.41 ± 0.26	1.27 ± 0.24	−13.40	0.001^*^
Alkaline phosphate	95.46 ± 31.88	99.40 ± 32.31	−2.92	0.003^*^
SGOT	21.99 ± 11.93	30.26 ± 19.57	−16.99	0.001^*^
ALT	20.09 ± 14.70	19.23 ± 17.04	−3.94	0.001^*^
Total protein	7.76 ± 0.57	8.00 ± 0.59	−8.84	0.001^*^
Total bilirubin	0.66 ± 0.54	0.66 ± 0.38	−0.02	0.983
Direct bilirubin	0.22 ± 0.20	0.23 ± 0.11	−3.58	0.001^*^
Indirect bilirubin	0.44 ± 0.45	0.43 ± 0.29	−1.37	0.170
GGT	21.60 ± 13.52	27.21 ± 40.60	−6.43	0.001^*^
HbA1C	5.98 ± 1.33	5.87 ± 1.15	−2.14	0.033^*^
RBS	125.06 ± 38.06	121.69 ± 32.84	−2.12	0.034^*^

All values are expressed in mean ± standard deviation. Statistical test used: Mann Whitney U test; ^*^P ≤ 0.05 is considered statistically significant.

### 3.4 Binary logistic regression findings

The binary logistic regression analysis revealed several significant associations between occupational status and various health conditions. Beedi rollers had substantially higher odds of experiencing back and leg pain (AOR: 3.137, 95% CI: 2.340–4.204), neck pain (AOR: 5.767, 95% CI: 4.200–7.920), and low back pain (AOR: 1.713, 95% CI: 1.270–2.312). Notably, respiratory symptoms such as sneezing (AOR: 3.130, 95% CI: 1.900–5.157) were significantly more common among beedi rollers. Eye-related problems, including redness of eyes (AOR: 2.193, 95% CI: 1.342–3.583), watering from the eyes (AOR: 1.979, 95% CI: 1.374–2.852), and decreased vision (AOR: 0.668, 95% CI: 0.492–0.908) also demonstrated significant associations. Symptoms like palpitation showed a particularly strong relationship (AOR: 7.023, 95% CI: 3.110–15.857). Dermatological issues such as pigmentation of hands (AOR: 3.339, 95% CI: 2.085–5.346) and itching of hands (AOR: 2.959, 95% CI: 1.641–5.334), along with chronic conditions like hypertension (AOR: 1.554, 95% CI: 1.165–2.073), further underline the occupational health burden in this population (Table 6).

**Table 6:** Binary Logistic Regression analysis of the health conditions for beedi rollers vs. non-beedi rollers.

Variables	Adjusted OR (95% CI)
Musculoskeletal problems	
Neck pain	5.767^***^ (4.200–7.920)
Elbow joint pain	0.557^***^ (0.389–0.797)
Low back pain	1.713^***^ (1.270–2.312)
Knee joint pain	0.686^**^ (0.524–0.899)
Back and leg pain	3.137^***^ (2.340–4.204)
Spinal pain	0.467^***^ (0.344–0.633)
Combination	0.443^***^ (0.310–0.632)
Respiratory problems	
Cough with sputum	0.252^***^ (0.127–0.502)
Nasal congestion	0.409^*^ (0.173–0.963)
Sneezing	3.130^***^ (1.900–5.157)
Ocular problems	
Ocular problem	1.705^***^ (1.246–2.333)
Decreased vision	0.668^**^ (0.492–0.908)
Watering from the eyes	1.979^***^ (1.374–2.852)
Redness of eyes	2.193^**^ (1.342–3.583)
Symptoms	
Weakness	0.526^***^ (0.405–0.682)
Breathlessness on exertion	0.495^**^ (0.315–0.777)
Palpitation	7.023^***^ (3.110–15.857)
Dermatological problems	
Pigmentation of hands	3.339^***^ (2.085–5.346)
Itching of hands	2.959^***^ (1.641–5.334)
Chronic disease	
Hypertension	1.554^**^ (1.165–2.073)

OR, Odds Ratio; CI, Confidence Interval; Odds ratio values are expressed along with 95% CI (in parentheses). Odds ratio adjusted for age, gender and education. Level of significance: ^*^P ≤ 0.05; ^**^P ≤ 0.005; ^***^p ≤ 0.001. Reference category for all independent variables is “No” (coded as 2); odds ratios represent the likelihood for “Yes” (coded as 1) compared to “No.” Dependent variable reference category: Non-beedi rollers.

Multivariable binary logistic regression (Table 7) revealed distinct biochemical and anthropometric differences between beedi rollers and non-beedi rollers. Elevated cotinine levels (AOR: 4.612; 95% CI: 4.007–5.309; *p* < 0.001) confirmed markedly higher nicotine exposure among beedi workers. Conversely, several biochemical parameters such as creatinine, SGOT, total protein, GGT and anemia status showed inverse associations with exposure, suggesting subtle early alterations in hepatic, renal, and nutritional status despite values often remaining within conventional reference ranges. A normal waist-to-hip ratio was more likely among beedi rollers (AOR: 0.298; 95% CI: 0.220–0.403; *p* < 0.001), while elevated HbA1c and blood glucose showed non-significant positive trends.

**Table 7:** Binary Logistic regression analysis of metabolic and biochemical health parameters for Beedi rollers vs. Non-beedi rollers.

Health Parameters	Adjusted OR (95% CI)
Cotinine levels	
Normal	Ref. (1.00)
High	4.612^***^ (4.007–5.309)
Creatinine levels	
Normal	Ref. (1.00)
High	0.532^***^ (0.393–0.721)
SGOT	
Normal	Ref. (1.00)
High	0.251^***^ (0.169–0.373)
Total protein	
Normal	Ref. (1.00)
High	0.546^***^ (0.403–0.740)
HbA1C	
Non-diabetic	Ref. (1.00)
Elevated/Diabetic	1.317 (0.931–1.862)
Waist to hip ratio	
Normal	Ref. (1.00)
At risk	0.298^***^ (0.220–0.403)
GGT	
Normal	Ref. (1.00)
High	0.613^***^ (0.398–0.943)
Blood glucose level	
Normal	Ref. (1.00)
Elevated/high	1.317 (0.931–1.862)
Anemia status	
Normal	Ref. (1.00)
Anemic	0.535^***^ (0.403–0.710)

OR, Odds Ratio; CI, Confidence Interval; Ref, reference category. Odds ratio values are expressed along with 95% CI (in parenthesis). Odds ratio adjusted for age, gender and education. Statistical test used: Binary logistic regression. Level of significance: ^***^P ≤ 0.001. Reference category: Non-beedi rollers.

## 4 Discussion

This present cross-sectional study provides compelling evidence of the substantial health burden experienced by beedi rollers compared to their non-beedi counterparts. The findings reveal a complex pattern of occupational health hazards that extend beyond respiratory complications to encompass musculoskeletal, ocular, dermatological, and metabolic disorders. The elevated cotinine levels among beedi rollers, despite their non-smoking status, unequivocally demonstrate significant environmental tobacco exposure, validating the occupational nature of their health risks.

The gendered nature of beedi rolling, with majority of workers being female, reflects the traditional division of labor in this industry and highlights the vulnerability of women to occupational health hazards. This demographic pattern is consistent with other studies showing that beedi rolling is predominantly performed by women from economically disadvantaged communities, often as a supplementary income source (26, 30). Women demonstrate heightened susceptibility to tobacco dust exposure due to smaller lung capacity, with research indicating higher prevalence of restrictive lung disease and more pronounced forced vital capacity reductions compared to males (31). The study reveals concerning workplace environmental conditions among beedi rollers, with majority working inside their homes, predominantly in living rooms and some report of lack of cross-ventilation. These findings align with previous research documenting the home-based nature of beedi manufacturing and its associated health risks (32). The predominantly indoor work environment, combined with poor ventilation, creates ideal conditions for concentrated tobacco dust exposure, explaining the elevated cotinine levels observed. Similar working conditions have been documented in studies from other regions of India, where beedi rolling is predominantly a cottage industry characterized by inadequate workspace infrastructure and minimal occupational safety measures (30). The confined indoor spaces likely result in inadequate air exchange rates, leading to accumulation of particulate matter and volatile organic compounds released during tobacco handling and processing (33). Furthermore, the absence of proper ventilation systems prevents the dilution and removal of airborne tobacco particles, potentially increasing the risk of respiratory sensitization and chronic inflammatory responses among workers (34). The domestic setting of beedi production also poses additional concerns regarding secondary exposure to family members, particularly children and older adult individuals who may share the same living spaces, thereby extending the health impact beyond the immediate workforce to vulnerable household populations (33).

The relatively modest respiratory morbidity observed in this study, despite the high cotinine levels indicating substantial tobacco exposure, presents an intriguing aspect that warrants further exploration. The healthy worker effect may play a significant role, whereby individuals who develop severe respiratory symptoms may be compelled to leave the occupation, leaving behind a workforce that appears relatively healthier than the general population exposed to similar conditions (35). Additionally, underreporting of symptoms cannot be dismissed, as workers may minimize their health complaints due to economic dependence on the occupation, fear of job loss, or normalization of mild respiratory symptoms as an inevitable consequence of their work (36). Cultural factors and limited access to healthcare may also contribute to under recognition of respiratory symptoms, particularly in populations where seeking medical attention for non-acute conditions is uncommon (37). Furthermore, the cross-sectional nature of this study may not capture the full spectrum of respiratory effects, as chronic conditions may develop over longer exposure periods than represented in the sample.

The study revealed that beedi rollers showed substantially higher prevalence of back and leg pain, neck pain, and lower back pain. The adjusted odds ratios from logistic regression analysis further emphasize these associations, with beedi rollers having higher odds of experiencing back, leg and neck pain. These findings are consistent with ergonomic studies highlighting the physically demanding nature of beedi rolling, which requires prolonged sitting in awkward postures, repetitive hand movements, and sustained neck flexion (9, 38). The biomechanical stress imposed by the traditional beedi rolling posture or ergonomics creates a cascade of musculoskeletal pathologies, as workers typically maintain a cross-legged seated position on floor surfaces without ergonomic support for extended periods (39). The repetitive fine motor control required for tobacco leaf manipulation and rolling generates cumulative microtrauma to the cervical spine, thoracic outlet, and upper extremity musculature (40). Additionally, the sustained forward head posture and rounded shoulder positioning during work contributes to cervicothoracic junction dysfunction and increased intradiscal pressure in lumbar segments (41). The absence of proper workstation design and frequent postural variation further exacerbates these conditions, leading to chronic myofascial pain syndromes and progressive joint degeneration that significantly impacts workers' functional capacity and quality of life (40). Comparative studies from other regions have reported similar musculoskeletal problems among beedi workers. A study from Karnataka reported high prevalence of back pain and neck pain among female beedi rollers, attributing these to prolonged sitting on floors and repetitive motions (42). International research on similar occupational groups, such as tobacco leaf processors in developing countries, has documented comparable musculoskeletal burden, suggesting that the physical demands of tobacco-related cottage industries pose universal health risks regardless of geographic location (43).

The respiratory health impacts were less pronounced than anticipated in the current study, despite the significant tobacco dust exposure evidenced by elevated cotinine levels. While beedi rollers showed higher prevalence of difficulty in breathing and sneezing, other respiratory symptoms were not significantly elevated. This pattern may reflect the complex nature of tobacco dust exposure effects, which can manifest differently from direct smoking-related respiratory complications. The relatively preserved lung function (FEV1/FVC ratio) suggests that current assessment methods may not fully capture the subtle respiratory impacts of chronic tobacco dust exposure (8). The discordance between biomarker evidence of exposure and overt respiratory symptoms could indicate that occupational tobacco dust exposure primarily affects upper respiratory tract function rather than lower airway mechanics, or that adaptive physiological responses may initially mask subclinical changes (44). Additionally, the chronic nature of exposure may lead to gradual accommodation of respiratory symptoms, making workers less likely to report mild dyspnea or cough that has become normalized over time. More sensitive pulmonary function parameters, such as fractional exhaled nitric oxide (FeNO) or high-resolution computed tomography findings, might reveal early inflammatory changes or structural abnormalities not detected by conventional spirometry (45). The latent period for developing significant respiratory impairment from occupational dust exposure may also extend beyond the current study's observation timeframe, suggesting the need for longitudinal assessments to capture the full spectrum of health consequences.

The ocular health findings are particularly noteworthy, with beedi rollers showing significantly higher rates of decreased vision, eye watering, and eye redness. These symptoms likely result from direct irritation by tobacco dust particles and volatile compounds released during the rolling process. The fine particulate matter from tobacco leaves can cause mechanical irritation of the conjunctival and corneal surfaces, leading to inflammatory responses that manifest as hyperemia and excessive lacrimation (46). Additionally, the close-proximity nature of beedi rolling work may contribute to visual strain and accommodative stress, particularly given the inadequate lighting conditions commonly reported in home-based work environments (47).

The significantly higher prevalence of dermatological problems, including hand pigmentation and itching, reflects direct contact with tobacco leaves and associated chemicals. These findings are consistent with case studies from other tobacco-processing regions documenting skin irritation and pigmentation changes among exposed workers (30, 48). The observed pigmentation changes may result from nicotine absorption through the skin combined with prolonged exposure to tannins and other phenolic compounds naturally present in tobacco leaves, which can cause localized melanin deposition (49). Furthermore, the repetitive handling of tobacco materials may compromise the skin's barrier function, increasing susceptibility to contact dermatitis and facilitating transdermal absorption of tobacco alkaloids and processing chemicals (48).

The study reveals significant metabolic and biochemical alterations among beedi rollers, including significantly higher diastolic blood pressure, elevated HbA1c, and altered liver function parameters. The lower SGOT levels and modified protein metabolism patterns suggest potential adaptive responses to chronic tobacco exposure or nutritional stress (50). The significant association between normal waist-to-hip ratio and beedi work likely reflects the sedentary nature of floor-seated work, which may impact body fat distribution patterns differently than other occupational activities (51). The study demonstrated significantly elevated cotinine levels among beedi rollers, with concentrations nearly four times higher compared to non-beedi rollers, providing objective biochemical evidence of substantial tobacco exposure in this occupational group. Cotinine, the primary metabolite of nicotine with a half-life of 16–20 h, serves as the gold standard biomarker for tobacco exposure assessment due to its stability and specificity (52). These elevated cotinine concentrations carry significant health implications, as chronic nicotine exposure has been associated with increased cardiovascular risk, including endothelial dysfunction, accelerated atherosclerosis, and heightened susceptibility to thrombotic events (53, 54). Furthermore, sustained cotinine elevation may indicate concurrent exposure to other tobacco-derived carcinogens and toxicants, potentially increasing the risk of developing tobacco-related malignancies even in the absence of active smoking (55). The four-fold increase in cotinine levels suggests that occupational tobacco dust exposure in beedi manufacturing may result in nicotine absorption comparable to light-to-moderate active smoking, thereby conferring similar health risks despite the different exposure mechanism. These metabolic findings are particularly significant given the limited existing research on biochemical impacts of tobacco dust exposure in occupational settings. While studies on active tobacco smoking have documented extensive metabolic effects, the specific biochemical profile of environmental tobacco exposure in beedi manufacturing represents a relatively unexplored area of occupational health research.

The elevated diastolic blood pressure and increased prevalence of hypertension observed among beedi rollers may be attributed to chronic nicotine absorption through dermal and respiratory routes, as nicotine is known to stimulate sympathetic nervous system activity and promote vasoconstriction, leading to sustained increases in vascular resistance (56). The higher HbA1c levels could indicate impaired glucose metabolism, potentially mediated by nicotine's effects on insulin sensitivity and pancreatic beta-cell function, similar to mechanisms observed in active smokers but possibly manifesting through different exposure pathways (57). The altered liver enzyme profiles may reflect hepatic adaptation to processing absorbed tobacco alkaloids and other xenobiotics, as the liver serves as the primary site for nicotine metabolism through cytochrome P450 enzymes (58). The unique occupational exposure pattern in beedi manufacturing, characterized by sustained low-level multi-route absorption rather than acute high-dose exposure, may result in distinct metabolic adaptations that warrant further investigation through longitudinal studies.

### 4.1 Limitations of the study and future directions

Certain limitations should be acknowledged when interpreting these findings. The cross-sectional design precludes establishment of temporal relationships between occupational exposure and health outcomes. The self-reported nature of many health symptoms may introduce recall or reporting bias especially for sensitive health outcomes, though the inclusion of objective biochemical markers strengthens the validity of findings. Given the large number of outcomes assessed, the possibility of type I error due to multiple comparisons cannot be ruled out. Potential clustering at village levels and residual confounding, including passive household tobacco exposure, cannot be excluded. The use of a forward stepwise variable selection approach may introduce model instability and potential overfitting, and thus the findings should be interpreted with caution despite adjustment for key confounders.

Future research should prioritize longitudinal studies to establish causal relationships between beedi rolling and health outcomes, with particular attention to the progression of musculoskeletal disorders and metabolic alterations over time. Detailed ergonomic assessments combined with intervention studies could provide valuable insights for developing targeted occupational health interventions. The unique biochemical profile observed in this study warrants further mechanistic research to understand the pathophysiology of environmental tobacco exposure in occupational settings. The findings of this study have important policy implications. The demonstrated multisystem morbidity and biomarker-confirmed occupational tobacco exposure provide evidence supporting the consideration of beedi rolling under hazardous occupation classifications. From a Universal Health Coverage perspective, these findings highlight opportunities to expand the reach of preventive, promotive, and occupational health services to informal workers.

Intervention research focusing on workplace modifications, ergonomic improvements, and personal protective equipment specifically designed for home-based beedi manufacturing could address the identified health risks while respecting the economic realities of this informal sector. Based on the significant occupational health hazards identified among beedi rollers, immediate implementation of specific ergonomic interventions is recommended, such as adjustable work tables replace floor-seated positions, neck and shoulder stretching exercises, and installation of window-mounted exhaust fans with face masks and cotton gloves to reduce tobacco dust exposure. Comprehensive annual health screening protocols should include cotinine level monitoring, musculoskeletal assessments, and cardiovascular risk evaluation. Community-based interventions should focus on health clinics providing specialized occupational health services and peer health educator training programs tailor made to this population. Policy measures may consider inclusion of beedi rolling in the hazardous occupation list and establishment of permissible tobacco dust exposure limits.

## 5 Conclusion

This study demonstrates that beedi rolling is associated with a significant and measurable occupational health burden, including elevated musculoskeletal morbidity, ocular and dermatological conditions, and biomarker-confirmed tobacco exposure reflected by higher cotinine levels. The predominantly home-based work environment with inadequate ventilation further amplifies these risks. These findings support the recognition of beedi rolling as a hazardous occupation and the implementation of targeted interventions, including ergonomic modifications, exposure reduction strategies, and routine occupational health screening within primary healthcare systems. Strengthening and expanding policy frameworks to include this workforce within occupational health protections presents a meaningful opportunity to advance Universal Health Coverage and promote health equity for this population.

## Data Availability

The raw data supporting the conclusions of this article will be made available by the authors, without undue reservation.
